# Relationship Between Stressful Life Events and Sleep Quality: Rumination as a Mediator and Resilience as a Moderator

**DOI:** 10.3389/fpsyt.2019.00348

**Published:** 2019-05-27

**Authors:** Yukun Li, Simeng Gu, Zhutao Wang, Hongfan Li, Xiayue Xu, Huan Zhu, Shiji Deng, Xianjun Ma, Guangkui Feng, Fushun Wang, Jason H. Huang

**Affiliations:** ^1^Department of Psychology, School of Medicine, Jiangsu University, Zhenjiang, China; ^2^Department of Encephalopathy, Lianyungang Affiliated Hospital, Nanjing University of Chinese Medicine, Lianyungang, China; ^3^School of Psychology, Institute of Emotional Studies, Nanjing University of Chinese Medicine, Nanjing, China; ^4^Department of Neurosurgery, Baylor Scott & White Health, Temple, TX, United States

**Keywords:** stressful life events, sleep quality, psychological rumination, psychological resilience, college student

## Abstract

**Purpose:** The aim of this study was to investigate the relationship between stressful life events and sleep quality and to probe the role of rumination and resilience in the relationship.

**Method:** The Adolescent Self-Rating Life Events Checklist, Ruminative Responses Scale, Connor–Davidson Resilience Scale, and Pittsburgh Sleep Quality Index were used among 1,065 college students. Statistical Product and Service Solutions (SPSS) 20.0 and the SPSS macro Process, which were specifically developed for assessing complex models including both mediators and moderators, were used to analyze the data.

**Results:** High scores of stressful life events predicted worse sleep quality. Rumination partially mediated the relations between stressful life events and sleep quality. Resilience moderated the direct and indirect paths leading from stressful life events to sleep quality.

**Conclusions:** The results demonstrate that stressful life events can directly affect the sleep quality of college students and indirectly through rumination. Additionally, increasing psychological resilience could decrease both the direct effect and the indirect effect of stressful life events affecting sleep quality. The results of this study may contribute to a better understanding of the effects, as well as the paths and conditions, of stressful life events on sleep quality in college students. Moreover, these findings can provide constructive suggestions for improving college students’ sleep quality.

## Introduction

There is little doubt that sufficient, restorative sleep plays a critical role in maintaining one’s physical and mental health. Poor sleep is considered as a predictive sign and symptom for many diseases and is associated with substantial decrements in life quality (1–4). College students are in a special period of life development, and good sleep is significantly important for them. However, it is reported that 12.9% to 52.8% of college students in China have sleep problems (5, 6). Troubled sleep has caused decrements in academic performance and increased risk-taking behaviors, and it has also increased the risk for subsequent declines in social, psychological, physical, and mental health. Therefore, it is important to identify and characterize the factors that modulate sleep quality and quantity.

A substantial body of literature has established the links between stressful events, negative consequences (7–10), and subsequent poor sleep quality (11–13). Stressful events refer to the things that compel people to make changes in their ongoing life patterns (14). Previous reports suggested that two important factors—rumination and resilience—are involved in psychological and physiological changes after stressful events. For example, Laura Blackburn’s study found that combat exposure affects soldiers’ posttraumatic stress disorder (PTSD) severity through intrusive and deliberate rumination, and resilience moderated the effect (15). Wu and his colleague examined the role of resilience and rumination in traumatic outcome and found that earthquake exposure-induced PTSD symptoms (including sleep disturbance) were modulated by brooding rumination and depression-related rumination (16). However, the roles of rumination and resilience have not been examined in the context of daily stressful events. Hence, we probe into the effects of stressful life events on sleep quality of college students in China and also the modulating roles of rumination and resilience in the process. We will introduce a new model about the mechanism of poor sleep quality in the context of daily stressful life events by examining factors that might predict poor sleep quality as well as those that might moderate the relationship.

### Rumination

According to the appraisal theory of emotion, emotions result from people’s interpretations and explanations of their circumstances. Richard Lazarus suggested that there are two major types of appraisals during stressful events (17): the first appraisal being automatic, unconscious, and fast activating; and the second appraisal being conscious and concerned with coping. Then there will be a reappraisal, which might be rumination. Rumination can be emotion-focused or problem-focused (18, 19) and can be defined as a response style whereby an individual tends to repeatedly think about the problematic situations or events, and focus on negative emotions and symptoms the adversity evoked (20–22). Previous studies indicated that rumination could induce many physiological changes, such as troubled sleep.

Harvey’s cognitive model of insomnia provides a framework to explain how rumination is linked to sleep disorders, especially at problematic situations (23). It is suggested that individuals with a ruminative response style tend to negatively tone cognitive activity to trigger both autonomic arousal and emotional distress. It is proposed that this anxious state focuses selective attention toward internal and external sleep-related threat cues. The unfortunate consequence is that the excessive and escalating anxiety may culminate in a real deﬁcit in sleep.

Some of the literature provides preliminary support for the model that rumination is associated with sleep problems. For example, Yang’s meta-analysis found that rumination is associated with deficits in core executive functions (EFs) (24), which have been suggested to be closely related to sleep problems among young adults with histories of suicide attempts (25). A high level of rumination is also related with worse sleep quality in teenage participants (26, 27). Studies also indicate that rumination may serve as a potential mediator in the relationship between stressful life events and later sleep-related difficulties (28, 29). For example, Zoccola and his colleagues first used portable sleep monitors to measure objective sleep-onset latency, and they found that poststressor rumination could positively predict longer sleep-onset latency (30). Taken together, these results demonstrate correlative relationships between stressful life events and rumination, and between rumination and later sleep-related difficulties. However, no clear pathway leading from rumination to poor sleep quality, especially in problematic situations, has been found.

Accordingly, we expect that daily stressful life events will predict relative increases in college students’ rumination. Furthermore, we expect that rumination will predict relative decreases in college students’ sleep quality. Thus, our hypothesis is: Daily stressful life events will positively predict poor sleep quality, through rumination (Hypothesis 1).

### Resilience

Resilience is a kind of ability to manage stressful events, and appears to have promise in buffering against adversity (31). Resilience can also be seen as an effective operation of the self-adjustment system (32). Why do some resilient individuals recover relatively quickly after a stressful event, returning to baseline functioning, whereas others are still struggling and having sleep difficulties? According to the meta-model, psychological resilience-related variables influence risk factors at multiple stages, from an individual’s appraisal of risk, his/her metacognitions in response to felt emotions, to his/her selection of coping strategies. It has been found to serve a protective role against physical and mental health problems. Furthermore, as stated in Kumpfer’s theory of resilience framework (33), the positive resiliency process could foster resilient reintegration, and individuals achieve a higher level of resilience after such a process. As a result, constant amplification of the protective effect promotes the virtuous circle of resilience.

Empirical research has provided strong support for the above theories. For example, several studies have suggested that psychological resilience can alleviate the adverse effects of traumatic childhood experiences on depression and reduce the risk of posttraumatic stress disorder (PTSD) while promoting posttraumatic growth (PTG) (16, 34, 35). A recent study by Houpy also found that resilience training is helpful for medical students to cope with occupational stress during the clinical year (36). Pietrzak’s team further found that people with high levels of resilience tend to have more psychological resources, which can be used to overcome adversity and adapt to changes in daily life (37). According to the protective mechanism of psychosocial resilience proposed by Rutter (38), resilience may play a protective role in reducing negative chain reactions, etc.

Therefore, we expect that resilience might moderate the adverse effects in daily stressful life events. Hence, our second hypothesis is: The effects whereby daily stressful life events positively affect poor sleep quality through rumination are moderated by resilience. More specifically, the impacts of daily stressful life events and rumination on sleep quality are expected to be decreased in the context of higher levels of resilience and to be increased in the context of lower levels of resilience (Hypothesis 2).

**Figure 1:**
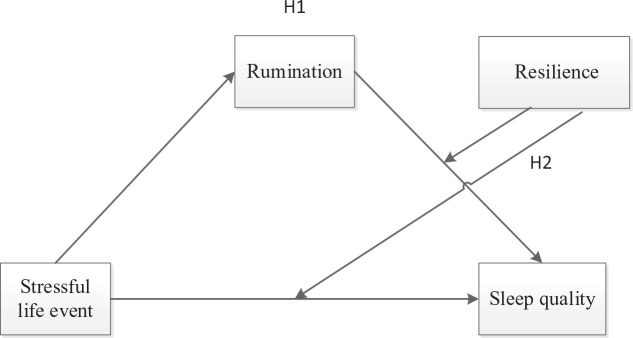
Conceptual model.

In this study, we sought to build on and extend prior studies on the relationship between stressful events and college students’ well-being in two ways: first, examining direct and indirect pathways, in order to provide an understanding of how stressful events affect sleep quality in college students’ daily life; second, investigating whether these pathways differ between individuals with different levels of resilience. Future prevention and intervention programs can be used to subsequently target their efforts at those students at risk. Our conceptual model in this study is as in **Figure 1**.

## Method

### Participants

A total of 1,065 college students from Jiangsu Province in China participated in this study (mean age = 20.21 years; SD = 1.35 years). These included 308 (28.92%) freshmen, 349 (32.76%) sophomores, and 408 (38.31%) juniors and seniors, consisting of 529 boys (49.67%) and 536 girls (50.33%) from different majors, including science and engineering (41.97%), medicine (38.22%), and liberal arts (19.81%). The exclusion criteria for subjects are average Symptom Check List 90 (SCL-90) scores >1.66, which means poor mental health, and students who failed the validity check items in the survey were excluded.

### Measures

#### Stressful Life Events

The intensity and frequency of participants’ stressful life events were evaluated with the Adolescent Self-Rating Life Events Checklist (ASLEC) (39), which includes 27 items grouped under six factors: interpersonal, learning stress, punishment, loss, health adaptation, and “other.” Each item is rated on a 6-point Likert-type scale. Participants were told to recall whether such events had occurred during the preceding 12 months of their lives. If they answered “no,” the score was 0; when answering “yes,” they were required to assess the impact of the stressful life event from 1 (not at all) to 5 (very much). ASLEC has been widely used in previous studies, has also been demonstrated to have good reliability and validity, and can be used to evaluate stressful events among teens, especially students in middle and high school and students in university. In this study, the Cronbach’s alpha of the ASLEC was 0.88 for the whole scale.

#### Rumination

Rumination was measured by the Ruminative Responses Scale (RRS), which is a subscale of the Response Styles Questionnaire (RSQ) (40). The RRS includes 22 items that describe the response to depression or other symptoms that arise from stressful events: Each item is rated on a 4-point Likert-type scale (from 1 = “almost never” to 4 = “almost always”). The RRS is composed of three dimensions, which are brooding, reﬂection, and depression-related. The Chinese version of RRS has been widely used and has reported good reliability and validity among Chinese college students (41). In this study, the Cronbach’s alpha of the RRS was 0.81 for the whole scale.

#### Resilience

Resilience was measured by the Connor and Davidson Resilience Scale (CD-RISC) (42), which measures personal qualities that enable people to thrive after exposure to stress and trauma. The CD-RISC involves 25 items and ﬁve dimensions. Each item is rated on a 5-point Likert-type scale (from 0 = “not true at all” to 4 = “true nearly all the time”), with higher scores indicating a stronger degree of resilience. The instrument’s reliability and validity is further evidenced by widespread use of the CD-RISC in research on Chinese students (43). In this study, the Cronbach’s alpha of the CD-RISC was 0.79.

#### Sleep Quality

Sleep quality and disturbance was measured by the Pittsburgh Sleep Quality Index (PSQI) (44), which includes 19 items and 7 factors, including sleep duration, sleep disturbance, sleep latency, daytime dysfunction due to sleepiness, sleep efficiency, overall sleep quality, and sleep medication use. Each of the factors yields a score ranging from 0 to 3. These 7 factors’ scores are summed to yield a total score ranging from 0 to 21. Higher score indicates worse sleep quality. The PSQI has been widely used in Chinese college students’ samples and shows great reliability and validity (45). In this study, the Cronbach’s alpha of the PSQI was 0.74.

### Procedures

The paper-and-pencil questionnaires were administrated to classes of 30–60 students during several data collection events within 1 week. We avoided the beginning of the semester, midterms, and finals, which are the periods that include more stressful events and may induce poorer sleep quality. Participants were guaranteed anonymity of their responses and confidentiality of the data. Completing the entire packet of instruments typically required 25–30 min. After data collection, SPSS 20.0 and the SPSS macro PROCESS (46), which were specifically developed for assessing complex models including mediators and moderators, were used to analyze the data. The study protocol conformed to the ethical guidelines of Jiangsu University and was approved by the institutional ethics committee, and written informed consent was obtained from each participant.

## Results

### Preliminary Analyses

In order to evaluate the common method variance in this study, we ran the Harman’s single-factor test. The results showed that no single factor can explain the majority of variance (the maximum component explained only 29.46% of total variance), which means that there was no common method bias in this study.

The means, standard deviations, and correlations for the measured variables are shown in **Table 1**. Data were first checked for normality, with a critical assumption underlying the asymptotically distribution-free procedure being used in the analysis. All results in this study indicated univariate normality for all measured variables.

**Table 1:** Means, standard deviations, and correlations among variables.

	Mean	SD	1	2	3
1. Sleep Quality	6.36	3.07	–		
2. Rumination	48.75	11.73	0.19**	–	
3. Resilience	58.90	16.81	−0.46**	−0.03	–
4. Stressful Life Events	45.09	12.87	0.18**	0.57**	−0.12**

n = 1065. **P < 0.01.

The average score on the PSQI was 6.36 ± 3.07. Of the 1,065 participants, 358 (33.62%) subjects (177 males, 181 females) met the criteria of sleep disturbance in China (PSQI scores ≥8) (47). The independent sample *t*-test of PSQI scores showed no statistically significant difference according to gender (*t* = 0.19, *P* = 0.85), and the one-way ANOVA showed no statistically significant differences in PSQI scores in terms of major (*F* = 1.15, *P* = 0.31).

Correlation analysis showed that stressful life events were positively correlated with PSQI scores and rumination. Rumination had a positive correlation with the PSQI scores. There was a negative correlation between resilience and PSQI scores (with higher PSQI scores indicating worse sleep quality).

### Mediation Analyses

We examined the indirect effects of rumination on the associations between stressful life events and sleep quality using the PROCESS procedure in SPSS 20.0 (46). The results are shown in **Table 2**, which indicated that stressful life events can positively predict sleep quality; when treating stressful life events and rumination as predictors, their effects on sleep quality were both significant. We created 1,000 bootstrap samples to further test the indirect effects of rumination. The results showed that the indirect effect was statistically significant (indirect effect = 0.08, 95% CI = 0.03–0.12), and the ratio of indirect to total effect of stressful life events on sleep quality was 41.10%. These results indicated that rumination serves a partial mediating function in the relation between stressful life events and sleep quality.

**Table 2:** Multiple regression analyses of the indirect effect of rumination.

Regression		Significance of regression coefficient			
Dependent variable	Independent variable	*β*	LLCI	ULCI	*t*
Sleep quality	Stressful Life events	0.18	0.12	0.24	6.01**
	Gender	0.03	−0.09	0.16	0.54
	Major	−0.05	−0.11	0.02	−1.47
	Grade	−0.02	−0.08	0.04	−0.63
Rumination	Stressful Life events	0.57	0.52	0.62	22.39**
	Gender	−0.08	−0.18	0.02	−1.53
	Major	0.03	−0.03	0.09	0.95
	Grade	−0.03	−0.08	0.02	−1.23
Sleep quality	Rumination	0.13	0.06	0.20	3.62**
	Stressful Life events	0.11	0.04	0.18	2.93**
	Gender	0.04	−0.08	0.17	0.72
	Major	−0.05	−0.12	0.01	−1.58
	Grade	−0.02	−0.08	0.05	−0.50

n = 1,065. **P < 0.01. Continuous variables were normalized. LLCI, lower level for confidence interval; ULCI, upper level for confidence interval.

### Moderation Analysis

A PROCESS analysis was used to test whether resilience moderates the links leading from stressful life events to sleep quality through rumination (**Table 3**), and the results showed that the standardized regression coefficient (β) of “resilience × rumination” remained significant; when resilience and rumination were controlled for, the same was true of “resilience × stressful life events.” This implies that the effect of stressful life events and rumination on sleep quality decreased as the level of resilience increased. In other words, the direct and indirect effects of stressful life events on sleep quality are moderated by resilience.

**Table 3:** Multiple regression analyses of the moderate effect of resilience.

Regression		Fit index		Significance of regression coefficient			
Dependent variable	Independent variable	*R* ^2^	F	*β*	LLCI	ULCI	*t*
Sleep quality	Resilience	0.34	92.81**	−0.39	−0.44	−0.34	−15.40**
	Rumination			0.19	0.14	0.24	7.49**
	Resilience × Rumination			−0.27	−0.32	−0.23	−12.77**
	Gender			0.01	−0.09	0.11	0.25
	Major			−0.01	−0.07	0.04	−0.49
	Grade			−0.01	−0.06	0.04	−0.38
							
Sleep quality	Resilience	0.29	70.49**	−0.39	−0.44	−0.34	−14.50**
	Stressful Life Events			0.12	0.07	0.17	4.49**
	Resilience × Stressful Life Events			−0.22	−0.26	−0.17	−9.19**
	Gender			0.03	−0.08	0.13	0.49
	Major			−0.01	−0.07	0.05	−0.38
	Grade			−0.02	−0.07	0.04	−0.59

n = 1,065. **P < 0.01. Continuous variables were centered at their means.

**Figure 2:**
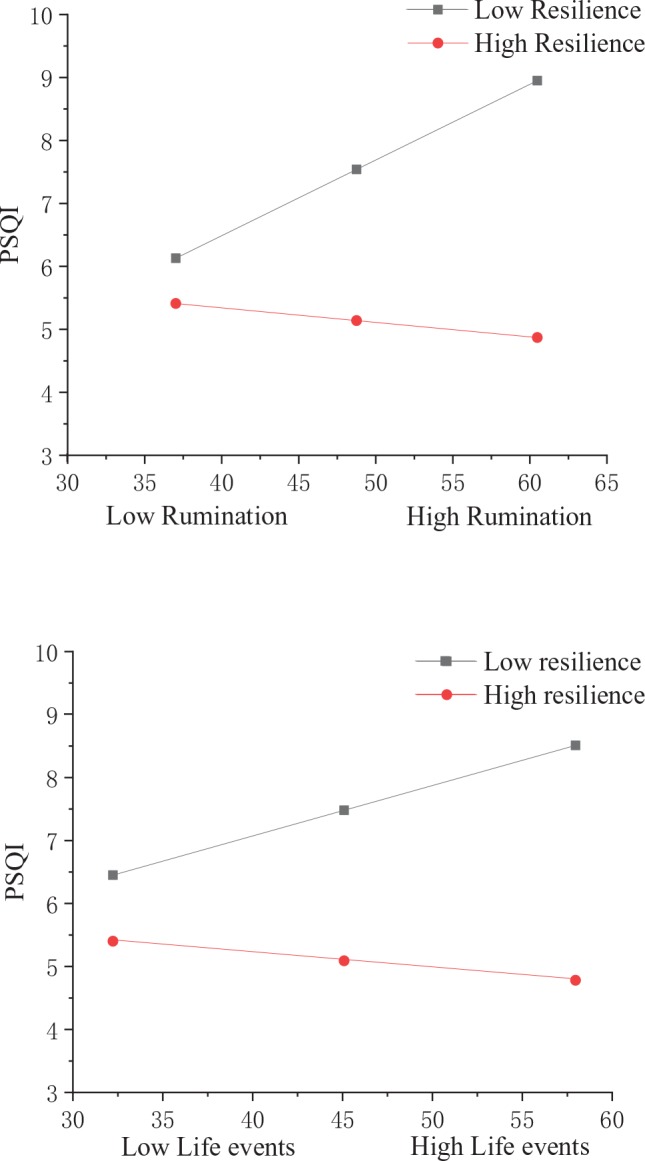
Simple slope analyses of the moderating effect of psychological resilience.

The nature of the moderation was further explored using a simple slope analysis (48) and conditioned at one SD above and below the mean; the results are shown in Table 4 and **Figure 2**. Compared with individuals who have higher levels of psychological resilience, individuals with lower levels of psychological resilience tend to show a stronger positive relationship between stressful life events/rumination and PSQI score.

**Table 4:** The direct and indirect effects at different levels of resilience.

Resilience	Effect		*LLCI*	*ULCI*
*M−SD*	Direct effect	0.03	0.01	0.04
	Indirect effect	0.05	0.04	0.07
*M*	Direct effect	0.01	−0.01	0.02
	Indirect effect	0.02	0.01	0.03
*M+SD*	Direct effect	−0.01	−0.02	0.02
	Indirect effect	−0.01	−0.02	0.01

LLCI, lower for confidence interval; ULCI, upper level for confidence level.

**Figure 3:**
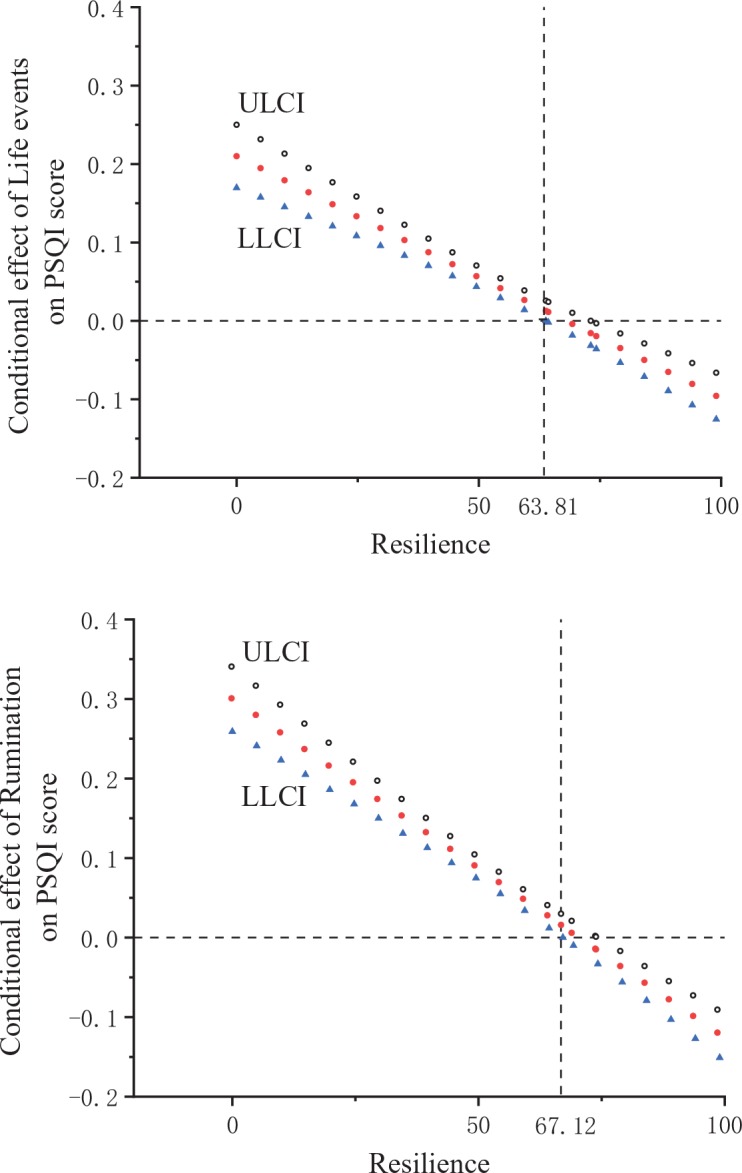
Visual representation of the conditional effect of stressful life events/rumination on sleep quality as moderated by resilience (LLCI, lower level for confidence interval; ULCI, upper level for confidence interval).

We tested the statistical insignificance of the moderating effect of psychological resilience on the association between stressful life events/rumination and sleep quality using the Johnson–Neyman (J-N) technique provided by the PROCESS procedure (46). The results are shown in **Figure 3**. Following the J-N technique, the 95% confidence interval of the conditional effect containing 0 indicates that the association was lost. As shown in **Figure 3**, with increasing resilience, the conditional effect of stressful life events on sleep quality declined dramatically and became statistically insignificant for students scoring above 63.81, as did the conditional effect of rumination when scores were higher than 67.12.

## Discussion

This study aimed to explore the relationship between stressful life events and poor sleep quality, and also the mediating and moderating factors between the bivariate links. We proposed that daily stressful life events positively predict poor sleep quality through rumination, and resilience moderates the links. Data from 1,065 college students in the Jiangsu Province of China supported our model. The results have several theoretical and practical implications, which we consider in turn.

First, this study sheds light on the nature of the relationship between daily stressful life events and sleep quality. Our study shows that college students who have experienced more stressful life events have poorer sleep quality, which is consistent with previous reports (12, 13, 49, 50). Psychophysiological research has provided a possible explanation that stress caused by stressful life events may lead to greater activation of the locus coeruleus norepinephrine (LC-NE) system and the hypothalamic–pituitary–adrenocortical (HPA) axis, which can increase excitability and aggravate difficulty in falling asleep (51). It also offers experimental data for our hypothesis about the neuromodulator basis of core affect. We propose that there are four basic emotions—fear, anger, happiness, and sadness—that are associated with three core affects: reward (happiness), punishment (sadness), and stress (fear and anger) (52). These three core affects are analogous to the three primary colors (red, yellow, and blue) and are subsided by three monoamines [DA (Dopamine) NE (Norepinephrine) 5-HT (5-hydroxytryptamine)]. This new model is called the “*three primary color model of basic emotions*.” (17) The activation of NE release by stressful events can antagonize the activity of 5-HT, which is the major substance associated with sleep.

Moreover, our study illuminates rumination as a mechanism that accounts for the relationship between stressful life events and poor sleep quality. According to Morin’s hyperarousal theory of insomnia (53), cognitive arousal may play an important role in sleep turbulence (54). Individuals with higher levels of rumination are more likely to have negative reappraisals of the past, which can increase cognitive arousal and consequently lower sleep quality. The result also offers some proof for our “emotional flow” theory. In this theory, we proposed that when something unexpected occurs, we first feel scared (fear) and then try to control the fearful situation (anger) (52), which could lead to a “fight” response. Afterwards, we might feel happy when successfully coping with the stressful event, or we may feel sad if we failed to cope (55). This kind of emotional flow constitutes emotions in sequence: fear–anger–happiness/sadness. According to Lazarus, fear and anger caused by stressful events are related to appraisal, while happiness or sadness is related to reappraisal, which might be manifested as rumination and affects other outcome variables, such as troubled sleep.

Finally, the data also show that the effects that daily stressful life events positively affect poor sleep quality through rumination are moderated by resilience. More specifically, the impact leading from daily stressful life events and rumination to sleep quality is expected to be decreased in the context of a higher level of resilience and be increased in the context of a lower level of resilience. Furthermore, according to the result of the J-N technique, resilience plays a significant moderating role in the majority of college students. The negative impact of stressful life events upon sleep quality declines with the increase of psychological resilience, and the effect may even disappear in highly resilient individuals (resilience scores greater than 63.81 and 67.12, respectively).

These new findings could reveal how stressful life events influence sleep quality and provide enlightenment for controlling sleep-related risk factors. Given the important influence of stressful life events on sleep quality, parents and teachers should pay attention to students’ stress levels. Methods of reducing stress can help create a supportive environment and improve sleep quality. In addition, our study suggested that rumination worsens sleep quality in those suffering from stressful life events. Hence, interventions like mindfulness training and positive reevaluation training would be beneficial for students with sleep problems. Finally, considering that resilience acts as a buffer between stressful events and sleep quality as well as the relationship between rumination and sleep problems, it is feasible to solve sleeping problems by improving psychological resilience level.

## Limitation and Future Research Directions

Although our data provide new evidence pertaining to the mediating effect of rumination and moderating effect of resilience on the relationship between stressful life events and sleep quality, our results should be assessed with care to include the background of the limitations inherent in our study. First, we collected data at one point in time, which limits the conclusions that can be made regarding the causal order of relationships. Hence, we would plan a second wave of data collection next year to substantiate the causality of our hypotheses. Also, experimental or longitudinal research designs from other researchers are welcome. Second, we relied on individuals’ self-reports for all variables in our model, which raises the concern of possible common method bias. However, our statistical analyses revealed that common method bias did not cause a major concern in our study. Therefore, we encourage future researchers to collect data from multiple sources to investigate our findings further. Moreover, sleep quality can be affected by many other factors, such as anxiety, depression, adult attachment, etc.; all these factors could serve as potential mediators in the stressful events–sleep relationship. Thus, multiple mediator models are suggested for future studies.

In conclusion, our study has contributed to debates about the role of stressful events in influencing sleep disturbances through the development and testing of a moderated mediation model. We introduced rumination and resilience as two key variables in this causal chain. We found that rumination partially mediated the relationship between daily stressful events and poor sleep quality in college students. We further found that resilience moderated the links. The findings are consistent with Lazarus’s appraisal theory of emotion and our own emotional flow theory. From our study, we can make a conclusion that college students’ sleep quality can be predicted by stress from day-to-day working lives directly, and sleep quality can also be predicted indirectly through ruminative response style. Increasing psychological resilience could decrease both the direct effect and the indirect effect that stressful life events affect sleep quality. Our findings suggest that resilience and rumination play very important roles not only in traumatic events but also in daily stressful events context. Our data reveal how stressful life events influence sleep quality and provide enlightenment for controlling sleep-related risk factors.

## Ethics Statement

The study protocol conformed to the ethical guidelines of Jiangsu University and was approved by the institutional ethics committee. Informed consent was obtained from each participant.

## Author Contributions

SG, FW, GF, and JH designed the experiment. YL, ZW, SD, and HL collected the questionnaire. HZ, XM, and XX did the data analysis. YL, SG, and FW wrote the paper.

## Conflict of Interest Statement

The authors declare that the research was conducted in the absence of any commercial or financial relationships that could be construed as a potential conflict of interest.
