# Supplementary material for: Relationship Between Stressful Life Events and Sleep Quality: Rumination as a Mediator and Resilience as a Moderator
Source: Front Psychiatry. 2019 May 27;10:348. doi: 10.3389/fpsyt.2019.00348 (PMC6545794; doi:10.3389/fpsyt.2019.00348)
Supplement: Supplementary file 1 [file Table_1.docx]

# Supplementary Information

### Supplementary Table 1. Multiple Regression Analyses of the Moderate Effect of Resilience on relationship between Stressful Life Events and Rumination

| Regression | |  | Fit Index | |  | Significance of Regression Coefficient | | | |
| --- | --- | --- | --- | --- | --- | --- | --- | --- | --- |
| Dependent Variable | Independent Variable |  | *R ^2^* | *F* |  | *β* | LLCI | ULCI | *t* |
| Rumination | Resilience |  | 0.16 | 27.83** |  | -0.06 | -0.20 | 0.09 | -0.77 |
|  | Stressful Life Events |  |  |  |  | 0.27 | 0.09 | 0.46 | 2.88** |
|  | Resilience× Stressful Life Events |  |  |  |  | 0.01 | -0.01 | 0.01 | 1.00 |
|  | Gender |  |  |  |  | -0.40 | -1.79 | 0.99 | -0.57 |
|  | Major |  |  |  |  | 0.24 | -0.53 | 1.01 | 0.61 |
|  | Grade |  |  |  |  | -0.97 | -1.71 | -0.23 | -2.58** |

#### Note: n =1065. ** P < 0.01. Continuous variables were centered at their means.
